# Pathology of the Nervous System in Von Hippel-Lindau Disease

**DOI:** 10.15586/jkcvhl.2015.35

**Published:** 2015-06-11

**Authors:** Alexander O. Vortmeyer, Ahmed K. Alomari

**Affiliations:** Yale School of Medicine, Department of Pathology, Division of Neuropathology, Connecticut, USA.

## Abstract

Von Hippel-Lindau (VHL) disease is a tumor syndrome that frequently involves the central nervous system (CNS). It is caused by germline mutation of the VHL gene. Subsequent VHL inactivation in selected cells is followed by numerous well-characterized molecular consequences, in particular, activation and stabilization of hypoxia-inducible factors HIF1 and HIF2. The link between VHL gene inactivation and tumorigenesis remains poorly understood. Hemangioblastomas are the most common manifestation in the CNS; however, CNS invasion by VHL disease-associated endolymphatic sac tumors or metastatic renal cancer also occur, and their differentiation from primary hemangioblastoma may be challenging. Finally, in this review, we present recent morphologic insights on the developmental concept of VHL tumorigenesis which is best explained by pathologic persistence of temporary embryonic progenitor cells.

## Introduction

Von Hippel–Lindau (VHL) disease is an autosomal dominant disorder that is caused by germline mutations in the tumor suppressor gene VHL on chromosome 3p25 (1, 2). It is characterized by frequent development of selective tumor types in specific topographic locations. Among others, central nervous system (CNS) hemangioblastoma and clear cell renal cell carcinoma (RCC) are the most consistently encountered tumors (3).

Tumorigenesis in VHL syndrome is linked to the loss of VHL tumor suppressor protein function in cell differentiation (4–7).The loss of protein function is the result of a second genetic “hit” which inactivates the wild type VHL allele (8). Subsequent to VHL gene inactivation and loss of VHL protein function, hypoxia-inducible factors (HIF1 and HIF2) are activated (9–11). HIF1 is a heterodimer transcription factor composed of HIF1á and HIF1â subunits (12). Through conditional transcriptional activation of effectors controlling vascularization, glucose metabolism and cell differentiation, HIF1 plays an important role in cellular response to oxygen changes (12). HIF2á (also known as endothelial PAS domain-containing protein -1 (EPAS1)) is another hypoxia-inducible factor that functions as an integral component of transcription factors involved in the body response to low oxygen level (13). In comparison to HIF1á which is expressed ubiquitously, HIF2á has a restricted tissue distribution and is expressed primarily in the vasculature of early developing embryo and subsequently in the lung, kidney interstitial cells, liver parenchyma, and neural crest cells (14).

VHL protein is part of a multiprotein complex that targets HIFá subunits for ubiquitin-mediated degradation in proteasomes (11, 15–19). In hypoxic conditions or with VHL biallelic inactivation, both HIF1á and HIF2á subunits will evade degradation, accumulate in the cell and complex with other subunits to form functional proteins (9, 10, 20). The consequences of HIF1á and HIF2á up-regulation are multifaceted. One major change includes the transcriptional activation of genes containing hypoxia responsive elements. VEGF is among the target genes that will be overexpressed leading to enhanced angiogenesis and oxygen delivery (12, 21). Additionally, HIF2á up-regulation is linked to enhanced dysregulated erythropoiesis while HIF2á deletion has been shown to result in anemia associated with decreased expression of erythropoietin (22, 23).

Despite the significant alterations at the cellular and molecular levels in association with VHL silencing, it has remained unclear, however, why loss of VHL function and subsequent HIF activation lead to tumorigenesis. Molecular and structural analogies of tumor cells with developmental tissues (6, 14, 24, 25) have led to revisitation of the hypothesis of a developmental origin of VHL disease-associated tumors (26, 27). There is increasing evidence that the “second hit” causes loss of pivotal VHL function during organ development leading to maldeveloped structures that represent prerequisites for tumor formation (28).

In VHL disease, the CNS is predominantly affected by hemangioblastic tumorigenesis, but may also be involved by endolymphatic sac tumors (ELST) or metastatic disease.

## Hemangioblastomas

Hemangioblastomas of the nervous system are present in 80% of VHL patients and represent a defining feature of VHL syndrome (3, 29). Topographic distribution of these tumors show a consistent pattern with the retina, cerebellum, brainstem and dorsal spinal cord being the most frequently involved locations (3). With recent advancements in sensitive imaging techniques, there has been a shift in the distribution of these tumors with increasing proportion of spinal tumors in comparison to the pre MRI era (30). Supratentorial tumors represent less than 3% of all tumors in most series (30, 31). In contrast to sporadic hemangioblastomas, VHL patients frequently have multiple hemangioblastomas, as is the case in 127 out of 160 patients in one report (30).

### Clinical and radiological presentation

**Figure 1.**
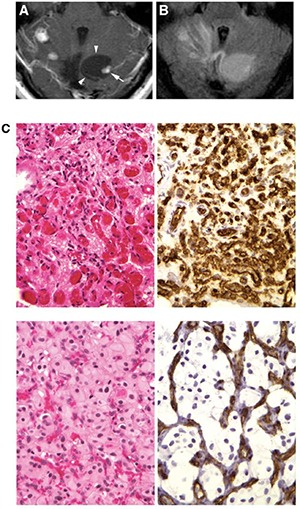
Radiological and histologic presentation of hemangioblastomas. **A,** T1-weighted postcontrast MR image shows right cerebellar hemangioblastoma (arrow) with an associated cyst (arrowheads); **B,** corresponding precontrast, fluid-attenuated inversion recovery, MR image demonstrating cyst hyperintensity (from Lonser et al., Ann Neurol 2005;58:392-399). **C,** Hemangioblastoma may present with mesenchymal (upper) and epitheloid structures (lower); Immunohistochemistry for vascular antigen CD34 reveals reactive vascularization (upper and lower right), neoplastic cells are negative for anti-CD34 (from Shively et al., J Pathol 2008;216:514-20).

Hemangioblastomas are benign tumors with no metastatic potential. However, as with other space occupying lesions of the brain, they may cause neurological deficit and carry a significant mortality rate if left untreated due to hydrocephalus, tonsillar herniation and brainstem compression (29, 32). Not infrequently, these tumors have an associated pseudocyst or syrinx evident by magnetic resonance imaging (MRI) (33) **(Figure 1)**. In addition to this cystic component, which may be much larger than the tumor itself, these tumors have a typical MRI appearance of densely contrast-enhancing solid mass with smooth margins (30, 34, 35).

### Pathological features

The gross appearance of these tumors commonly shows a solid nodule with an associated pseudocyst. Pseudocysts result from secretory tumoral activity (33) and disappear after successful surgical removal of the tumoral nodule (33). The tumoral nodule is usually soft with bright or dark red color, and occasional yellow areas seen upon sectioning (28). Histological findings vary greatly and are dependent on tumor size (25). There are two main cellular constituents of hemangioblastomas, “stromal” cells and vascular cells. By selective genetic analysis, “stromal” cells have been shown to be neoplastic cells (36, 37). The origin of the “stromal” cell has been controversial (38); however, most consistently proposed has been a hemangioblastic or hemangioblastic progenitor origin (27, 39–42). Since hemangioblasts were originally discovered in embryonal tissues almost 100 years ago (43–45) and are not known to exist in adult CNS tissue, the concept of a developmental origin of hemangioblastoma developed early (26, 27) and will be further discussed below.

The second cellular constituent of hemangioblastoma is represented by abundant vascular cells that show no evidence of biallelic VHL inactivation and are therefore mostly – if not entirely – reactive (36, 46). The most plausible explanation for the abundance of reactive vascular structures in hemangioblastoma is the tumoral “stromal” cell expression of angiogenic factors (47). However, whether a subset of vascular structures may represent differentiation products of neoplastic hemangioblastic “stromal” cells (vasculogenesis) has been investigated with controversial results.

Most immunohistochemical studies identify “stromal” cells and vascular cells as separate cytological constituents with no evidence of transition between the two cell types: they either report distinct immunoreactivity with different markers of interest (48–52) or find vascular markers factor VIII and/or factor XIIIa exclusively expressed in the vascular component of tumors (48, 53). In contrast, other studies reported expression of vascular markers in “stromal” cells (54–56). Cell culture studies either revealed no evidence of interconvertibility between endothelial cells and “stromal” cells (57) or evidence for vascular differentiation capacity of “stromal cells”(58). Microdissection studies revealed evidence for vasculogenesis in precursor structures of hemangioblastomas (59), but not in frank tumors (46).

Within hemangioblastomas, the proportion of “stromal” and vascular cells can vary greatly and has been used as the basis of the traditional morphologic sub-classification of hemangioblastoma into a vascular-rich reticular (also called “mesenchymal”) cell phenotype and a “stromal” cell-rich cellular (also called “epitheloid”) variant (60) **(Figure 1)**. A study of 156 variably-sized hemangioblastomas revealed strong correlation between histologic subtype and tumor size (25). In the aforementioned study, all tumors smaller than 8 mm^3^ showed exclusive reticular structure, also known as “angiomatotic”, or “mesenchymal”, that is characterized by small loosely scattered tumor cells separated by extensive vascularization (25). Larger tumors reveal additional phenotypic change, neoplastic stromal cells that are enlarged in size with abundant cytoplasm and large nuclei, and frequently clustered in groups (25). Cytologically, the stromal cells in these latter neoplasms may have an abundant glycogen or lipid-rich cytoplasm imparting a clear appearance of the cell on routine H&E sections. Foci of extramedullary erythropoiesis can be detected in cellular (epitheloid) areas, but are not detected in reticular (mesenchymal) tumor portions. It has therefore been postulated that reticular lesions represent an earlier stage of tumorigenesis from which epitheloid tumor with extramedullary erythropoiesis may develop (25). Interestingly, these morphologic changes within individual hemangioblastomas have previously been interpreted as different stages of embryonic hemangioblastic maturation (40).

## Endolymphatic Sac Tumors

The endolymphatic duct/sac system is composed of single-lumen thin long endolymphatic duct ending in a short pouch-like endolymphatic sac (61). The interior of this system is lined by single-layered cuboidal endolymphatic duct/sac epithelium (62). The intraossesous part of the endolymphatic duct/sac system is also referred to as the vestibular aqueduct (63) and represents the site of origin of ELST (64). The endolymphatic duct/sac system is part of the nonsensory membranous labyrinth of the inner ear with a potential functional role in maintenance of homeostasis and pressure of the inner ear, phagocytosis of debris and immunologic functions (65–68).

### Epidemiology

ELST was first established as a distinct pathologic entity by Heffner in 1989 (69). However, ELST had not been recognized as a component tumor of VHL disease until 1997, after the identification of 15 inner ear tumors in 13 out of 121 patients with VHL disease, while none of 253 patients without evidence of VHL disease had inner ear tumors (70). Additional studies documenting VHL inactivation by microdissection and PCR-based loss of heterozygosity analysis provided genetic and molecular confirmation of this association (64, 71, 72). Moreover, neoplastic cells were found to show activation of both HIF1and HIF2 as well as expression of CAIX and GLUT-1 which are downstream targets of HIF, and co-expression of EPO and EPOR, which has been implicated in promotion of tumor growth (64, 73). The prevalence of ELST in VHL patients has been reported to be approximately 10% to 15% based on imaging studies, with 30% of these patients having bilateral tumors (74).

### Clinical presentation

ELST can cause hearing loss, tinnitus, vertigo and facial nerve paresis (75). Hearing loss can occur in larger tumors with invasion of the otic capsule, but also in smaller tumors. The proposed mechanisms of hearing loss in these tumors include hemorrhage and/or endolymphatic hydrops (76, 77).

### Morphology

**Figure 2.**
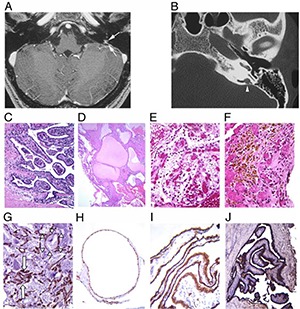
Radiologic, morphologic and immunohistochemical features of ELSTs. Serial magnetic resonance (MR) and computed tomography (CT)-imaging of the temporal region from a VHL patient with hearing loss demonstrating the development of a left endolymphatic sac tumor. (**A)** Axial, T1-weighted enhanced MR-imaging reveals enhancement in the region of the left endolymphatic duct (arrows). **(B)** Corresponding, axial, non-enhanced, CT of the left temporal bone shows bony erosion confined to the left endolymphatic duct (arrowhead). Morphologic spectrum of ELSTs: Papillary structures were observed in all tumors **(C),** whereas cystic areas **(D)** were seen in half of the cases. One tumor had areas of epitheloid clear cell clusters **(E),** reminiscent of clear cell renal carcinoma. Extensive hemosiderin deposits were evident in half of the tumors **(F).** A feature of all tumors was intensive vascularization (**G,** immunohistochemistry with anti-CD 34). Note the abundant vessels in papillary stroma (arrows) and the immediate contact of numerous small vessels with the cystic epithelium (arrowheads), which appears to be induced by expression of HIF and VEGF by the epithelial tumor cells. Immunohistochemistry for NSE **(H),** MAK6 **(I)** and AE1/AE3 **(J)** was consistently positive (from Glasker et al., Cancer Res 2005;65:10847-53).

ELSTs present grossly as bright or dark red soft tissue masses (76). The histological appearances of these tumors are variable and are usually composed of a mixture of three main architectural patterns; papillary, cystic and epithelioid clear cell (64, 69). One consistently observed feature is the presence of extensively vascularized papillary structures. These papillary structures are lined by a single row of cuboidal epithelial cells. Focal cystic growth can be observed in a subset of tumors as is focal clusters of epitheloid clear cell. The cysts have a single epithelial lining and frequently contain proteinaceous material. Mitotic figures are rare. Extensive hemosiderin deposits are common and associated with degenerative features including fibrosis, inflammation and cholesterol cleft formation **(Figure 2)**. Immunohistochemical analysis of VHL disease-associated ELSTs reveals positive immunoreactivity with anti-NSE, anti-MAK6, and anti-AE1/AE3 (64, 78). Immunohistochemistry for EMA and S100 is positive in subsets of cases (79). Some sporadic tumors have also been reported to be positive for GFAP and vimentin (78).

In addition to frank tumors, multifocal microscopic cystic and papillary structures were identified in the intra- and extra-osseous endolymphatic duct/sac system in VHL patients (64). These structure were different in their molecular profile from similar cystic proliferations identified in non VHL patients; in VHL patients, the cystic and papillary structures showed loss of the wild-type VHL allele in addition to positive nuclear staining for HIF1 and HIF2 and expression of target proteins CAIX and GLUT-1 (64, 80). It has been proposed that these structures represent tumor precursors (64).

## Metastasis

Metastasis into the nervous system can occur from three different types of VHL disease-associated tumors. Most frequently, metastasis is caused by RCC which often may show striking resemblance to primary hemangioblastoma or ELST. Far less frequently observed are metastatic pheochromocytoma / paraganglioma or metastatic neuroendocrine tumors.

### Metastatic renal clear cell carcinoma

Metastasis of RCC can occur anywhere within the nervous system. However, if metastasis occurs into cerebellum, brainstem, or spinal cord, differentiation from primary hemangioblastoma may constitute a diagnostic challenge. Both tumors share histologic features including cells with clear or vacuolated cytoplasm, extensive vascularization and occasional clustering of epitheloid cells. Additionally, both tumors share VHL gene deficiency as well as expression of VEGF, HIF, CAIX and other VHL target proteins. Several subtle morphological features have been proposed to help differentiate these tumors. Most importantly, cytoplasmic membranes are more distinct in renal cell carcinoma, while less well defined in hemangioblastoma cells. Also, identification of mitotic figures strongly favors metastatic renal cell carcinoma. Necrosis is virtually never seen in hemangioblastoma, unless tumors had been pretreated with radiation or embolization (28).

**Figure 3.**
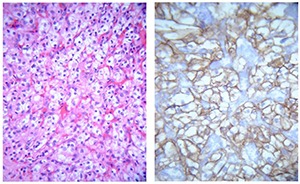
Metastatic RCC. Left, Metastatic renal cell carcinoma to brain resembling hemangioblastoma, H&E stain; right, immunohistochemistry for EMA shows characteristic membranous staining.

Immunohistochemistry is a valuable tool to distinguish hemangioblastoma from metastatic RCC. Epithelial membrane antigen (EMA) was originally described to selectively identify RCC (81, 82). However, focal expression of EMA may rarely occur in hemangioblastoma (83) **(Figure 3)**. Anti-inhibin A showed a high sensitivity and specificity for hemangioblastoma in one study (84). Other potentially useful markers include anti-CD10 for the identification of RCC and D2-40 and aquaporin-1 for the selective identification of hemangioblastoma (85–88). More recently, a combination of PAX-2 and PAX8 (positive in RCC) and inhibin A (positive in hemangioblastoma) has been proposed as the most useful panel of markers to resolve this differential diagnosis (89). Another important role of immunohistochemistry is the identification tumor heterogeneity that is caused by tumor metastasis into hemangioblastoma which is being reported in up to 8% of hemangioblastomas (90, 91).

## VHL tumorigenesis

### Development of tumors in VHL disease– Arvid Lindau’s hypothesis

Decades ago, the hypothesis of a hemangioblastic nature of CNS tumors in VHL disease fueled an even older debate on the origin of cancer which notably has not been settled until today. In the early and mid-1800s, Joseph Recamier and Robert Remak both noted that cancer tissue resembled embryonic tissue (92). In 1874–1875 Francesco Durante and Julius Cohnheim proposed cancer to originate from small collections of embryonal cells that persist and do not differentiate into mature adult tissue (92–94). In the early 1900s, this theory was rejected (92, 95, 96). When, however, Arvid Lindau discovered and described in detail the gross and histologic changes of VHL disease, he came to the conclusion that “…all types of neoplasia might be explained by a disturbed balance during mesodermal development during the third month of embryonal life” (26). While Arvid Lindau was therefore a strong supporter of the “embryonic rest”-hypothesis to explain CNS tumorigenesis in VHL disease, he subsequently noted that “an underlying early maldevelopment” may give rise to two different pathologic processes “Hemangioblastoma” and “capillary angiomatosis” (27). He was, however, unable to explain how “early maldevelopment” can produce such extreme neoplastic diversity: a tumor composed of hemangioblastic cells on one hand, and a tumor composed of mature vascular cells on the other.

### Lindau’s hypothesis – analytical and experimental evidence

More recently, we worked on a resolution of this seemingly contradictory nature of VHL disease-associated CNS tumors. Key strategy for this effort was to change the primary target of histologic analysis. Instead of analyzing tumor tissues, we analyzed normal-appearing tissues of VHL patients in which tumors are known to frequently occur, spinal cord and cerebellum.

**Figure 4.**
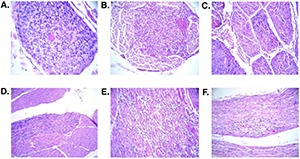
Developmentally arrested structural elements (DASEs), incidentally discovered in grossly normal-appearing nerve root tissue of VHL patients by histologic analysis of random spinal cord sections obtained at autopsy (from Vortmeyer et al., Ann. Neurol. 2004; 55:721-728). All depicted DASEs were present intradurally in different nerve roots. All lesions reveal circumscribed accumulations of persistent embryonal VHL-deficient hemangioblast progenitor cells (see references 24 and 98). **A,** cross-section of a nerve root, filled with embryonal cells; **B**, The central portion of the root shows an accumulation of embryonal cells which is sharply demarcated from surrounding normal root tissue; **C,** A longitudinal nerve root section reveals frequently observed longitudinal extension of DASE’s along fiber tracts; **D,** intraradicular DASE with embryonal cells, diffusely scattered around neurites; **E,** DASE, selectively involving a central nerve root fascicle; **F,** circumscribed DASE in nerve root; focal clear cell phenotype and early multifocal vascular perfusion may indicate early progression into hemangioblastoma.

The study of tumor-free central and peripheral nervous system tissue of VHL patients revealed numerous microscopic structures that are not encountered in similar tissues of non-VHL control patients (24, 64, 97, 98) **(Figure 4)**. In essence, these structures represent the “early embryonal maldevelopment” postulated by Lindau and have hence been designated “developmentally arrested structural elements” (DASEs) (97). DASEs represent precursors of CNS tumors in VHL disease; however, only a small number of DASEs progress to frank tumor during the lifetime of a VHL patient (24, 25, 97).

The key constituent of DASEs are small primitive VHL-deficient cells that apparently represent immature hemangioblast progenitor cells, accompanied by reactive vasculature (24, 97, 98) **(Figure 4)**. Early tumor arising from DASEs is highly vascularized and resembles “angiomatosis”. Upon further tumor growth, tumor cells increase in size, develop epitheloid architecture and may differentiate in blood (25, 42, 58). The morphologic sequence of tumorigenesis from DASEs to frank tumor reveals transition of mesenchymal cells into epitheloid structures and thus explains the diverse and complex histologic presentation of CNS tumor in VHL disease.

According to Knudson’s two hit hypothesis, tumorigenesis in VHL disease is initiated by the “second hit”, inactivation of the wild type copy of the VHL allele in patients that carry the VHL mutation (8). The recent identification of numerous VHL-deficient DASEs in tumor-free CNS tissues of VHL patients led to the conclusion that the “second hit” is necessary, but insufficient for tumorigenesis (98, 99). Instead, “second hits” appear to be capable of producing DASEs from which frank tumors may or may not develop. The nature of the third hit allowing DASEs to develop into frank tumor remains unknown.

### Persistence of temporary embryonal progenitor cells

In VHL disease, the embryonal rest hypothesis does not necessarily imply the generation of neoplastic tissue during organ development. Instead, it implies the causative genetic hit to occur during organ development with the effect of developmental arrest of variable numbers of embryonal/fetal cells that have the capacity of eventual neoplastic proliferation. Key arguments are:

a) Organ selectivity: Precursor and tumor formation occurs abundantly in selective tissue structures, and not at all in others; In VHL disease, there is dramatically increased risk of development of hemangioblastoma, renal clear cell carcinoma, cystadenoma of pancreas and endolymphatic sac, and neuroendocrine tumors, but no increased risk for any other type of cancer.

b) Tumor specificity: While large varieties of different tumors are known to exist in CNS and kidney, only tumors with specific histology occur in affected organs.

c) Consistent topographic distribution of precursor structures/tumors: Tissue development is strongly associated with pattern formation. VHL disease not only targets the nervous system, it does so in a near-predictable pattern of distribution. Neoplastic structures are frequently observed in spinal cord and cerebellum, only rarely in cerebrum. Within the cerebellum, the molecular layer is primarily affected. In spinal cord tissue, precursors/tumors are detected far more frequently in dorsal than in ventral roots. The most frequently affected specific topographic CNS site is the obex of the brainstem (E. Oldfield, personal communication).

d) Stem/progenitor cell properties of VHL deficient neoplastic cells: Multiple different analytic and/or experimental approaches have identified the VHL-deficient neoplastic cell as primitive hemangioblastic progenitor cell with capacity of differentiation into red blood cells and/or primitive vascular structures. Although not definitively demonstrated, there is strong supportive evidence for hemangioblastic differentiation to occur during brain (and even stronger evidence for kidney) development. Hemangioblastic cells are not part of the mature nervous system; instead, hemangioblastic cells persist in the nervous system due to pathologic events during tissue development and represent a condicio sine qua non for tumor development.

### Alternative hypotheses

Alternative hypotheses for tumor development include a) tumorigenesis from a pre-existent glial, neuronal, or mesenchymal cell (100); b) tumorigenesis from circulating cells (101); c) tumorigenesis from a stem cell (96, 102, 103).

a) Tumorigenesis from a pre-existent cell in the nervous system would imply dedifferentiation of mature cells into hemangioblastic progenitor cells which not only has never been demonstrated, but should be expected to be an extraordinarily complex molecular and structural process. Activation of HIF occurs in tissues under hypoxic stress, but is not known to be related to tumorigenesis. Structural analysis of precursor/tumor structures in the nervous system reveal the pathology to evolve from minute poorly differentiated foci into larger lesions with increased maturation (24), with erythropoiesis occurring only in largest-sized frank tumors (25). Simply, the abundance of minute structural changes in VHL tissues is explained more easily by an origin from VHL-deficient aberrant cells; furthermore, the most frequent occurrence of precursor structures in nerve root tissue provides a very limited set of candidate cells for tumor development.

b) Circulating bone marrow cells have been implicated in the generation of or contribution to brain tumors (101). This hypothesis would explain tumor specificity, but require bone marrow hematopoietic cells to be primarily targeted by the second hit. The VHL deficient hemangioblastic progenitor/stem cell is the most primitive form of hemangioblast, originally discovered in the yolk sac (45). To support this hypothesis, it would be necessary to demonstrate presence of these primitive hemangioblastic precursor cells in fetal or adult bone marrow. More difficult to explain, however, would be the principles of organ selectivity and topographic distribution of VHL tumors. In addition, bone marrow pathology is not known to exist in VHL disease. 

c) Since VHL deficient tumor cells in nervous system have been identified as hemangioblastic progenitor cells, an origin from VHL-deficient stem cells with hemangioblastic differentiation potential certainly deserves consideration. While the role of stem cells in the brain remains controversial, a stem cell origin of brain (and many other) tumors is being increasingly accepted (103). By definition, stem cells are totipotent cells with differentiation potential along multiple (in the brain along astrocytic, oligodendroglial and neuronal) cell lineages which is not the case in VHL disease (tumor specificity). Nevertheless, while the pathogenic cell in VHL nervous system remains best-characterized as VHL-deficient hemangioblastic progenitor cell, the second hit could occur in a totipotent stem cell, with VHL inactivation being exclusively permissive for hemangioblastic differentiation. However, the stem cell hypothesis fails to explain organ selectivity and consistent topographical lesion distribution within organs. Also, experimental knock-out of the VHL gene in cell systems has so far not revealed evidence for hemangioblastic differentiation.

In essence, stem cell hypothesis and embryonal rest hypothesis are not entirely exclusive. Like hematopoietic progenitor cells, stem cells are derived from embryonal cells. The key difference is the time of the second hit, VHL inactivation. The stem cell theory would allow for the second hit to occur any time, while the embryonic rest hypothesis postulates the second hit to occur early during tissue development, producing an early and pathologic set of VHL deficient hemangioblastic progenitor cells. It is the embryonic rest hypothesis which most comfortably explains the key arguments presented above.

This modified embryonal rest hypothesis has not been proven, but it most effortlessly explains clinical, pathologic and experimental evidence available so far. Similarly effortlessly it can be applied to any other classic tumor suppressor gene syndrome (e.g. MEN1, neurofibromatosis), with confirmatory literature on record. Thus, VHL disease is a naturally occurring human tumor suppressor gene “model” the unique features of which give insight into the biology of neoplastic disease and cancer.

## Conclusion

VHL disease is a classic tumor suppressor gene syndrome caused by VHL gene germline mutation. In the nervous system, the “second hit” - VHL wild-type gene inactivation – induces DASEs which have the potential to develop into benign hemangioblastic tumors. In the kidney, VHL-deficient cells give rise to renal clear cell carcinoma which can metastasize into brain and spinal cord. Endolymphatic sac tumors may develop in the vestibular aqueduct with the potential of brain invasion. While the molecular consequences of VHL inactivation have been worked out in detail, fundamental questions have remained unexplained by these studies. The fundamental questions include organ selectivity, tumor specificity, the consistent lesional distribution pattern, and the embryonal/stem cell nature of the tumor cells. These intriguing features are best explained by the “second hit” occurring during nervous system development. Application of this concept may generate new research approaches to VHL-deficient tumors, their sporadic counterparts as well as tumors in the context of other tumor suppressor gene syndromes.
